# Mechanical Properties of Hardened 3D Printed Concretes and Mortars—Development of a Consistent Experimental Characterization Strategy

**DOI:** 10.3390/ma14040752

**Published:** 2021-02-05

**Authors:** Maximilian Meurer, Martin Classen

**Affiliations:** 1 Institute of Structural Concrete, RWTH Aachen University, 52062 Aachen, Germany; 2 Department of Civil Engineering, Materials and Constructions, Katholieke Universiteit Leuven, 3000 Leuven, Belgium

**Keywords:** additive manufacturing, 3D-printing concrete (3DPC), digital production with concrete, compressive strength, tensile strength, shear strength, fracture energy, test setups

## Abstract

Today, it is already foreseeable that additive manufacturing of mortar and concrete has groundbreaking potential and will revolutionize or at least fundamentally change the way we build. In recent years, 3D concrete printing (3DCP) with extrusion-based deposition methods has been pushed forward by a growing research community. Albeit being regarded one of the most promising innovations in construction industry, a consistent characterization methodology for assessing the constitutive behavior of 3D printed, hardened cementitious materials is missing, so far, which hinders its widespread use in engineering practice. The major objective of this paper is to fill this gap by developing a new experimental framework that can thoroughly describe the mechanical properties of 3D printed cementitious materials. Based on both a review of state-of-the-art test setups and a comprehensive experimental campaign, the present paper proposes a set of easy-to-use experimental methods that allow us to assess flexural, tensile, shear and compressive strength as well as fracture energy of 3D printed concretes and mortars in a reliable and reproducible manner. The experimental results revealed anisotropic material behavior for flexural, tensile, shear and compressive loading. Furthermore, they confirm that interval time (time gap between deposition of subsequent layers) has a crucial effect on investigated material properties leading to a severe reduction in strength and fracture energy for longer interval times.

## 1. Introduction

Additive manufacturing (AM) or 3D-printing of concrete is an emerging technology that has high potential to contribute to further automation in the construction sector and eventually lead to fully digital fabrication of concrete structures. An increasing number of universities and companies is working on the development of extrusion-based printing systems, printable cementitious materials and concrete mixtures as well as methods for integration of reinforcement into the printing process.

Lighthouse projects as the printed cyclist bridge in Eindhoven [1], the DFAB house in Zurich [2] and a two-story building in Belgium [3] show the great potential and the possibilities of today’s digital concrete fabrication technology. A detailed differentiation of different digital concrete fabrication systems can be found in [4]. During the early stages of AM of concrete, the rheological requirements and new challenges in concrete mixture design owing to the absence of formwork as well as the antagonism of the new engineering terms printability and buildability were important research issues. The quantification of fresh concrete properties through new test methods [5,6,7,8,9,10] and initial models to describe the rheological properties required for stacking of layers [10,11,12] laid the foundation for further research. Different studies analyzed the influences of nozzle size, shape and, e.g., induced vibrations on the flow characteristics [13,14] and promoted a deeper understanding of the physics of extruded fresh concrete. Further, the used machinery and printing parameters such as printing speed and interval time between the application of the subsequent layers have been extensively investigated [15,16]. In addition, the integration of reinforcement is an important research topic. Different approaches either based on nailing layers together [17], printing the reinforcement in advance [2], or adding reinforcement simultaneously to the concrete printing [18,19,20] have already been investigated.

Today, there is already a large number of viable digital production methods available. However, the construction sector in Europe and other countries is regulated by a strict set of rules aligned to prevent injury or damage to persons and property caused by the built environment. The restrictive set of rules and the conservative design approaches resulting from this design philosophy are in stark contrast to the extremely progressive and forward-thinking research efforts in the field of digital production with concrete. This contrast is exemplified by the following fact: While many new digital construction methods, such as extrusion-based 3D concrete printing, rely entirely on the adhesive (tensile) strength of the layered connection along the interfaces, the design concept propagated in Europe and Western countries does not allow us, or only within narrow limits, to consider this tensile strength of concrete for calculating structural resistances. Obviously, verification of structural integrity and safety of printed structures is impossible within this current set of regulations. This conflict of objectives is further intensified by the fact that, due to the layering of filaments, it is in the nature of additively manufactured members to have a non-homogenous internal structure. The interfaces lead to an anisotropic behavior of hardened printed concrete on a macroscale, which needs to be additionally considered in designing printed members [14,21,22,23]. More than that, the tensile strength and mechanical properties such as, e.g., fracture energy of interface regions, are not fixed values but depend on time elapsed after application of two subsequent semi-fresh concrete layers [24].

In order to allow for making widespread use of groundbreaking successes made in the field of digital fabrication, we obviously need new impulses to reform our design strategy. The basis for developing new design concepts is formed by reproducible and reliable standard test methods for determining the mechanical properties of printed structures. Up to now, standard test methods known from concrete construction have been adopted without adapting them to the special boundary conditions of printed components. However, the layered structure of the material resulting from the new production methods requires the development of new experimental methods. This paper examines to what extent the adaption of well-known standard tests, e.g., for the investigation of tensile and bending behavior or fracture energy, is reasonable and adequate and which deficits arise when using these setups for printed concrete. Additionally, new test setups (e.g., for the investigation of shear along the interfaces) are proposed and assessed for their suitability.

Finally, the idea of a completely new testing method based on the use of a combined torsion-axial testing unit TORAX is presented, which allows us a systematical and reproducible determination of all relevant material properties of printed components by use of one single test setup [25].

## 2. Review of Current Characterization Methods for Constitutive Behavior of Hardened 3D Printed Concrete

The term 3D printed concrete or digital manufactured concrete is widely used in the ongoing research publications and industry. However, a closer look reveals that due to the propagated aggregate sizes, several printable concrete mixtures from the literature should be actually specified as mortars. The tests setups for printed cementitious materials, presented in this paper, are intended for analyzing both mortars and concretes with small aggregate sizes (<8 mm). For the reason of simplicity, the term “printed concrete” is thus used throughout the whole paper for all cementitious materials (concretes and mortars) with maximum aggregate diameter of 8 mm. For statements that are explicitly pointed towards cementitious materials with maximum aggregate size smaller than 4, the term mortar is used.

**Figure 1:**
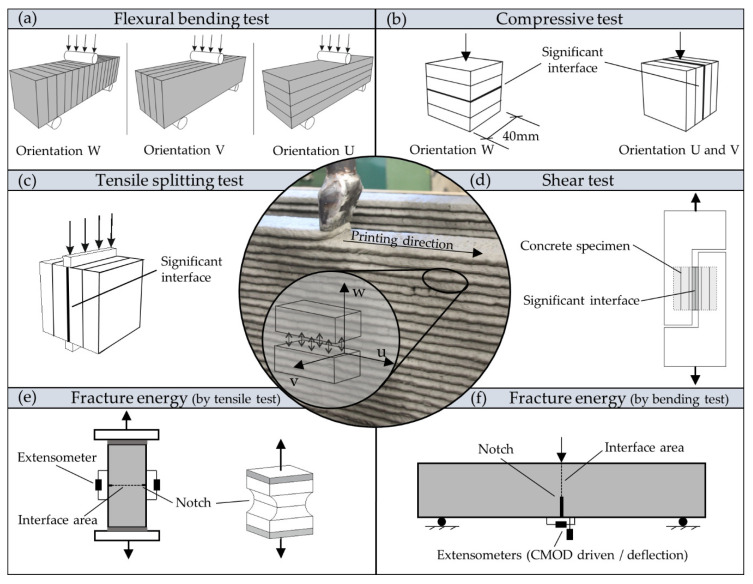
Compilation of different test methods on hardened printed concrete to investigate the mechanical properties during (**a**) flexural stress with 3-point bending tests [15]; (**b**) compressive stress test [15].; (**c**) Tensile test setup with tensile splitting test [11]; (**d**) Shear test setup [28]; determination of fracture energy with (**e**) uniaxial tensile test setup of dog-bone shaped specimen [24] and cylindrical specimen [22]; (**f**) 3-point bending tes.t setup [29].

In the recent literature several test methods for printed concrete were presented and anisotropic mechanical properties were found for different loading directions (in-plane, perpendicular to the printed interface or parallel to printed interface, out-of-plane). Initially, a new coordination system was introduced by Bos et al. [26] to take into account the printing direction and the interface arrangement as shown in the center of Figure 1**.**

Figure 1a illustrates 3-point flexural bending test arrangements from the literature. Here, an anisotropic behavior was determined for orientations U, V and W, with reduced strength for the interface loaded in mode I (perpendicular to the interface, orientation W) [14,27]. Depending on concrete mixture and printing parameters a general decrease in flexural strength compared to casted concrete was found in most flexural tests with W-orientation. In addition, the interval time, the delay between the deposition of subsequent layers, has been varied in some of these tests. For an interval time of around zero to one minute, Le et al. [22] and Rahul et al. [27] found about a 40% decrease, whereas Wolfs et al. [15] found only a 15% decrease and Zhang et al. [23] found nearly equal values of casted and printed specimens.

Compression tests in different directions showed only a slight anisotropic behavior, but a reduction of the compressive strength by 10% [27] and 25% [15] compared to casted concrete.

Further shear tests were conducted on the interface region by Rahul et al. [27] as shown in Figure 1c. A decrease in shear resistance in the interface region of 22.5 to 29% compared to casted concrete was found.

For the determination of the tensile strength either a tensile splitting test or direct tensile test is used as shown in Figure 1c,e. The tensile splitting test by Wolfs et al. [15] is a small-scale test of a standardized tensile splitting test, which creates tensile stresses perpendicular to the load plane with a relatively simple setup. Here, no anisotropic behavior was found in contrast to the 3-point bending test setup. Le et al. [22] performed tensile bond tests on cylindrical specimen and found a decrease of around 23% compared to casted concrete.

Even though most researchers used similar experimental principles to assess the material strength, the studies came to significantly different results. These discrepancies may be related to different test setup dimensions and small deviations in loading and supporting conditions. In addition, the scatter in observed mechanical properties is amplified by different printing parameters. Especially the fresh concrete production scheme may influence the mechanical properties [28]. When mixing concrete ingredients at the beginning of the printing event, printed specimens are likely to have lower strengths compared to specimens printed with continuously mixed fresh concrete.

Besides strength, also the toughness of concrete interfaces plays a major role to ensure sufficient bond and structural resistance of 3D printed structures [30]. Debonding of a printed interface is a quasi-brittle process. The printed interface is able to transfer residual bond stresses until a certain limit crack width is reached. To evaluate the toughness of the material, we can assess the fracture energy $G_{f}$, defined as the energy dissipated to fully open a crack. The determination of the fracture energy can for instance be done by a direct tensile test. Wolfs [24] performed direct tensile tests with rotating and non-rotation loading plates with dog-boned shaped specimen, pictured in Figure 1e. The non-rotation loading plates are able to uniformly distribute tensile stresses on the cross-section and also allow measuring the post-peak behavior, which enables the determination of fracture energy. The determined fracture energy of the interface region of 28.4 N/m is relatively low compared to plain concrete with similar mixture. Unfortunately, no values of the used casted concrete are available. Alternatively, a 3-point bending test can be used, as shown in Figure 1f. The 3-point bending test captivates with the simplicity of the test setup and is therefore much more robust and less prone to errors than a uniaxial tensile test. In general, a notch is needed to concentrate the stresses and achieve constant crack development [29,30]. However, different testing methods do not necessarily lead to same test results, as size of the specimens, support conditions, notch depth or slenderness [29,31] also have an important influence. This dependency causes a problem in recalculating and comparing different results. With regard to testing fracture energy in plain concrete, larger aggregate sizes and content enhance the fracture energy through more tortuous crack patterns [32]. The water–binder ratio (w/b) of the concrete mixture affects the fracture energy, as well. A lower water content reduces the porosity and increases the strength parameters of the cement paste. In consequence, the crack pattern tends to run through the aggregates in high strength concretes, instead of running around the aggregates. This goes along with a rather low dissipated energy and leads to a smoother crack surface on the macro-level [29,32]. For 3D printed concrete, these parameters have not been analyzed in detail. In general, a difference in the density of the matrix material and interface regions can be expected. The matrix material is more dense than ordinary casted concrete, whereas the interface area has significant density losses, which leads to reduced material strengths and weak points regarding the protection against chemical impact [22,27].

Further, the surface moisture content plays a major role in the inter-layer bonding strength. If the interfaces are not protected against drying, significant strength losses occur after 10 min, whereas protected interfaces are stable up to 90 min [11]. Wolfs et al. [15] found flexural strength losses of 50% for unprotected specimens compared to protected after 4 h.

Obviously, there is a strong need for objective and coherent test methods that enable a reproducible experimental determination of the complex material behavior of 3D printed concrete. The described developments indicate the complexity of testing hardened 3D printed concrete. Until now, there is no unified testing strategy that can fully characterize all relevant properties. There is a strong need for refined experimental methods focused on individual stress transfer effects within both, the printed interfaces and the layered composite material. Besides established test methods for the characterization of concrete behavior (e.g., standardized tensile, compressive and bending tests) the focus of this paper is on proposing entirely new characterization methods, that overcome the drawbacks of current ones. To this end, the current paper is organized as follows. After presenting the used machinery, materials, and production methods, different test setups and experimental results are explained in detail. Then, strengths and weaknesses of testing methods are evaluated with regard to suitability for characterization of 3D printed concrete, to finally propose an enhanced test method that allows characterizing several different mechanical properties of hardened 3D printed concrete in a unique, universal test setup.

## 3. Materials and Methods

### 3.1. Printing System and Concrete Mix Design

The experimental investigations on 3D printed concrete specimen described in the following were conducted at the Institute of Structural Concrete, RWTH Aachen University and Materials and Constructions group, KU Leuven. The temperature during the production, storing and testing was constant at 22 °C with a relative humidity of 50%. To investigate the mechanical properties of printed interface regions, a printing system and a printable concrete were initially developed, to achieve comparable results. The basis for the system is a Putzmeister S5 grout pump, which can run with different rotor and stator sizes. In order not to be limited by the pump when choosing the maximum grain size, a D8-1.5 soft auger was used. With this auger a maximum grain size of 5 mm and up to 40 L/min can be transported. As the handling system, a computerized numerical control (CNC) machine IMATEC FBF 2000 was used. The CNC can move in three axes. In the Y- and Z-axis the machine head is moving and in the X-axis the printing bed is moving. Structures up to 1500 mm length, 900 mm width and 1000 mm height can be realized with a maximum machine velocity of 1.5 m/min. The dry concrete components were mixed in advance and stored in dry and sealed buckets. At the beginning of a printing event, water is added to the dry mixture, which is then mixed for 90 s followed by a 30 s break and another 30 s of mixing. Then, the fresh concrete is filled into the reservoir of the pump, where a screw conveyer constantly moves the material and transports it to the auger. The concrete is transported from the pump around 2.5 m to the CNC machine in a 1-inch diameter hose.

Generally, the mechanical properties of printed concrete are influenced by different time dependent effects. One effect is the age of the fresh concrete mixture, another effect is the interval time between the deposition of subsequent concrete layers. To reasonably limit the number of tests presented in this paper, both effects have not been varied independently, but in a constant ratio: for all tests, the concrete age at application was chosen equal to the interval time.

Based on the machine setup, a printable mix was designed with a maximum grain size of 4 mm. To verify the fresh concrete properties, a Hägermann flow table (HFT) test according to DIN EN 1015-3 [33] was conducted. For pumpable and buildable concrete, Näther et al. [34] determined 10 cm (i.e., no deformation) in the first test and around 18 cm in the second measurement after 15 shocks. Tay et al. [35] proposes values between 15 and 19 cm for a good buildability and a smooth surface. Regarding the aforementioned experiences from the literature, a water–binder ratio of 0.43 was chosen, with 17.2 cm spread diameter after 15 shocks. In Table 1, the final concrete mixture is listed, and the sieve fractions are shown. Only a small ratio of 2–4 mm grain was chosen to ensure a dense structure and a good buildability. Nevertheless, to achieve a more cost-efficient and climate-friendly printing mixture, further material developments in reducing the water to binder ratio and increasing the maximum grain size are necessary.

**Figure 2:**
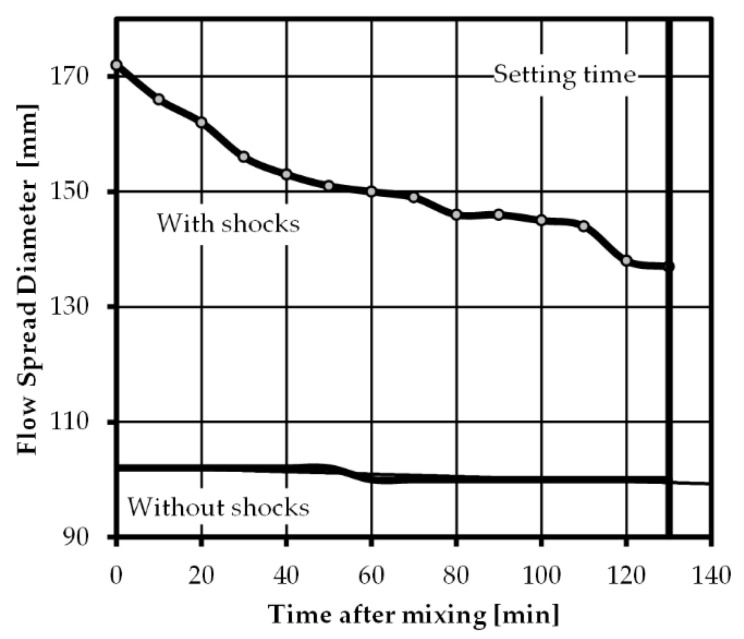
Development of spread diameter of Hägermann flow table test with increasing time after mixing solid components with water.

The behavior of fresh concrete over time, especially the balancing of pumpability and buildability is crucial in 3D printing of concrete. In order to quantify the fresh concrete properties of the proposed mix design over time, HFT [33] and Vicat test [36] according to the respective design codes were conducted on fresh concrete. In an interval of 10 min HFT measurements were recorded. The results are pictured in Figure 2 and show the decrease in flowability of the fresh concrete over time. Simultaneously, the setting time and end of setting time were determined by the Vicat apparatus. The setting time was reached after 135 min, which describes the end of the processability of the mixture. In addition, the standard stiffness was determined to be 8.25 mm in two separate measurements.

### 3.2. Printing of Large-Scale Demonstration Wall Segment

**Figure 3:**
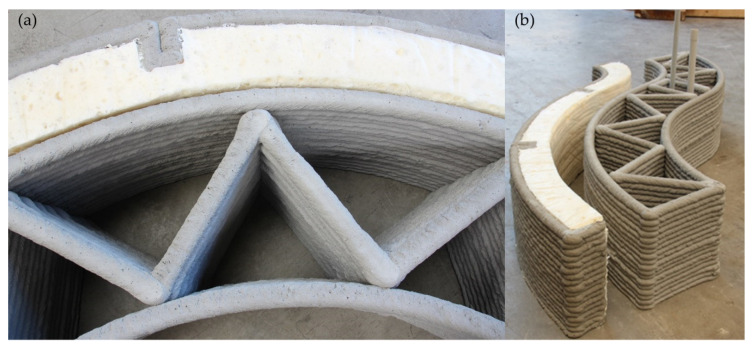
Printed demonstration object with integrated insulation and piping system printed at the Institute of Structural Concrete, RWTH Aachen University: (**a**) top view, (**b**) side view.

To practically test the presented 3D printing system and materials an exemplary wall segment was printed, as illustrated in Figure 3. The design of the demonstrator reveals the ability to produce free-form structures with reduced material consumption but structural performance that is comparable to ordinary masonry wall structures. For this purpose, an S-shaped wall segment with an inner truss structure was designed and printed. The aforementioned machinery and a round nozzle with 27 mm diameter were utilized. The inner structure allows for piping integration and provides enough space for thermal insulation. The S-shaped outer shell of the wall segment has a width of 250 mm and the inner truss pattern has a mean distance of 240 mm, a respective 120 mm per strut. With a printing speed of 1.5 m/min a satisfactory printing quality was achieved. The total height of 27 cm was realized without measurable deformation in the lower layers. Due to the successful printing, the concrete mixture as well as the printing system are considered as realistic testing environment (proof of concept).

### 3.3. Experimental Campaign and Production of Specimens

The goal of the following investigations is to systematically assess the suitability of different test setups for determination of interface properties of printed concrete with variable interval time between deposition of subsequent layers. An overview of testing methods, parameters investigated, and dimensions of specimens is given in Table 2.

Distinction is made between fully printed specimen and hybrid specimen, in which only the investigated filament was printed, while bulk material of the specimen was made of casted concrete.

**Figure 4:**
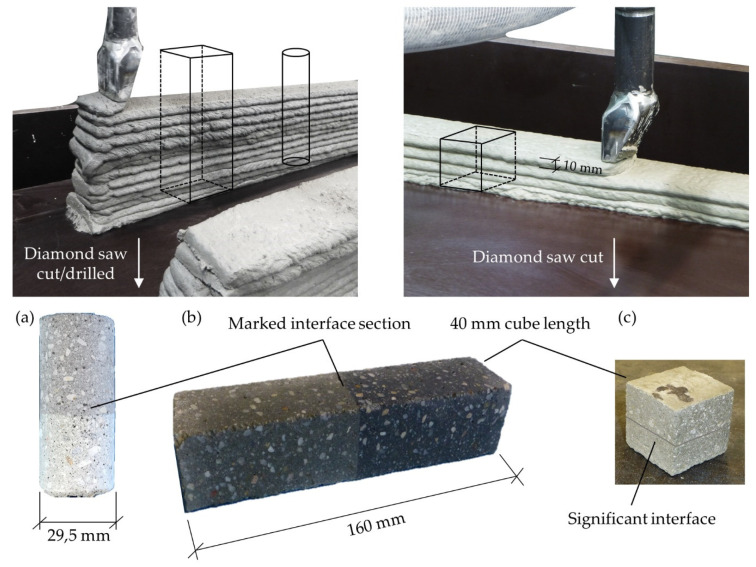
Production process of printed specimens; left for shear and bending experiments and right for tensile testing and bending in U- and V-direction. After curing the specimens were extracted for testing (**a**) shear strength, (**b**) flexural strength and (**c**) tensile strength.

The production of fully printed specimen was done using a 45 mm nozzle, as shown in Figure 4. Different specimens were printed for each time interval of 1, 10, 20, 30 and 40 min between the application of the next layer. A constant print speed of 1.5 m/min was set and all layers were printed in the same printing direction to ensure comparable interfaces. The printer head was lifted after finishing a layer and traversed to the respective starting point, which took 60 s. As shown in Figure 4, the interface regions were not protected from drying during the time between application of the next layer. Due to the high production effort, three hybrid specimens per time interval were produced. In setups with fully printed specimens four to five specimens were tested for each time interval.

The concrete age was chosen equal to the interval time. Compared to e.g., Li et al. [28] this leads to an enhanced time affected behavior, because both effects, concrete age and interval time influence the interface properties. This is a conservative assumption especially for larger structures with long printing paths and, e.g., the delivery of concrete by a mixing truck. The dehydration of the interface during the interval time is believed to cause the decreased properties found by different authors [11]. Specimens for bending and shear tests were printed with two different concrete colors to highlight the position of the printed interface and the bond behavior of layers. For this purpose, black pigments were added into the concrete mixture after printing the first layer of the tested interface.

After curing the specimens were cut by a diamond saw into the desired specimen dimensions, listed in Table 2 and shown in Figure 4. Reference mold-cast specimens were produced on every printing event to exclude aberrations due to material inconsistency.

**Figure 5:**
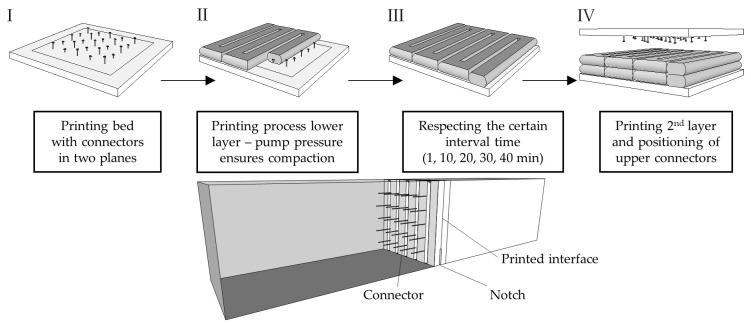
Schematic depiction of production of hybrid specimens with 150 mm edge length and nail connection to self-compacting concrete.

In addition, hybrid specimens with printed filament and casted bulk material have been produced for determination of fracture energy in larger 3-point bending tests. The intention was to reduce the production effort through just printing the investigated interface regions of 150 × 150 mm² with the respective interval times and connecting them to casted concrete to form the outer parts of the beam specimen. Figure 5 shows the production process starting with the printing bed, which is prepared with protruding steel connectors, sticking in styrofoam plates. The connectors were arranged symmetrically in two levels with 10 and 5 mm height to prevent failure in one plane. Afterwards, the connectors were overprinted by filaments of 27 mm width and 15 mm height. Due to the pump pressure the whole connector was well embedded with concrete. After waiting for the particular interval time, the second layer was printed in the same pattern and connectors were inserted into the fresh concrete on the top through a styrofoam plate to create a symmetrical specimen. After curing of the printed interface, the specimens were arranged in the center of a formwork with 700 mm length and 150 mm cube length to cast the outer parts of the bending beam. After curing the printed interface section in the center position of the beam was notched 3 cm.

### 3.4. Testing Procedure

#### 3.4.1. Flexural Bending Test—Unnotched

The flexural strength in orientation W and V is determined according to European standard EN 196-1 [37]. This requires unnotched specimen sizes of 40 mm height, 40 mm width and around 160 mm length. The span width between the fixed bearing supports is 100 mm and the load is applied through a roller support in the center. The test was performed load driven by a PistonStroke Controller TT1184 while the maximum load was measured to determine the flexural strength.

#### 3.4.2. Flexural Bending Test—Notched

**Figure 6:**
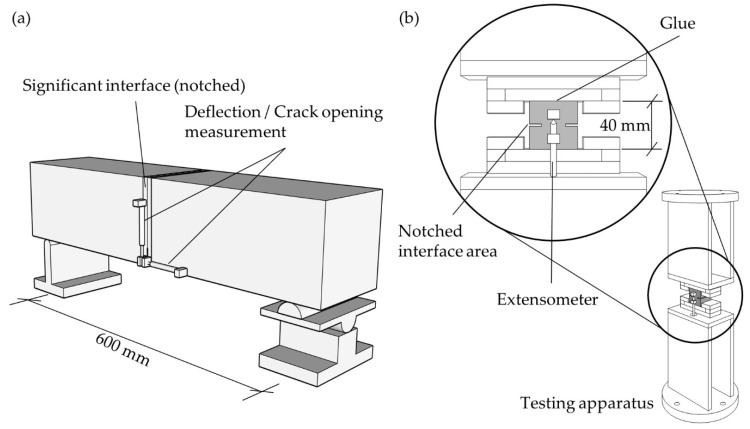
(**a**) Three-point bending test setup for specimen with 150 mm edge length and 700 mm length and (**b**) Uniaxial tensile test setup.

Hybrid printed/casted specimen with 150 mm height, 150 mm width and 700 mm length (Figure 6, dimensions according to German Committee for Reinforced Concrete for steel fiber-reinforced concrete) were tested under 3-point bending with an l/d-ratio of 4. The setup is shown in Figure 6a, with the load application in the center position above the 30 mm notch. The load was applied through a roller support in the center, above the printed interface. Through free rotation in longitudinal and transversal directions, possible torsion stresses are avoided. The tests were performed displacement controlled with a testing velocity of 0.1 µm/s until peak load. After reaching the peak load, the velocity was adjusted to maximum 1 µm/s to reduce the test duration and to avoid distinct viscoelastic behavior. The investigations were performed by an Instron 5582 universal tester The deflection is measured by transducers. In order to eliminate measurement aberrations due to tilting of the beam, the deflection and crack opening were measured on both sides of the beam as shown in Figure 6. The proposed testing methods enable us to measure the post-peak behavior, until the applied load tends to zero and the beam fails under its self-weight. Evaluation of the post-peak branch allows us to assess the fracture energy of the printed interface region.

#### 3.4.3. Tensile Test

In order to determine the fracture energy in a more direct way than with flexural bending tests, a uniaxial tensile test setup was designed to measure the post-peak behavior while determining the tensile strength. The designed test setup is illustrated in Figure 6b. To allow measuring the post-peak response of the specimens, a deformation-controlled, closed-loop testing procedure is required.

Closed-loop testing systems provide the ability to directly control the deformation of a loaded specimen. This considerably enhances the precision, stability, and scope of the experiments compared to conventional testing procedures with monotonic load increase. Closed-loop machines equipped with servo hydraulic control can be used to determine a stable response of a test specimen even in the post-peak branch by monitoring and controlling the physical quantities that are sensitive to its behavior. Such testing procedures are essential for determining the post-peak response of a tensile specimen. The test setup is highlighted in Figure 6b. The extensometers are glued on the specimen and measure the displacement in the notched area on a length of 10 mm. After formation of a crack, the measured displacement corresponds to the crack mouth displacement (CMOD), which is used as feed-in signal for the deformation control.

Besides the closed-loop testing technique, the stiffness of the test setup has to be significantly higher than that of the specimens. Thus, the setup was designed in a way, that all connections can be assumed rigid within the expected load range of less than 10 kN. The two parts of the system are mounted with 20 mm thick base plates to the bottom and the load cell of the test frame. Although the test area is quite small, the whole setup is around 50 cm tall. This is caused by requirements due to machine dimensions and required space to mount the extensometers to the specimen. An Instron 5582 universal tester with a maximum load of 100 kN was used as the testing device.

In order to recognize induced bending stresses due to imperfections, the extensometers are glued on the front and back side of the specimen. The specimen itself is glued in between two steel plates with HBM X60 2-component fast curing glue.

#### 3.4.4. Shear Test

**Figure 7:**
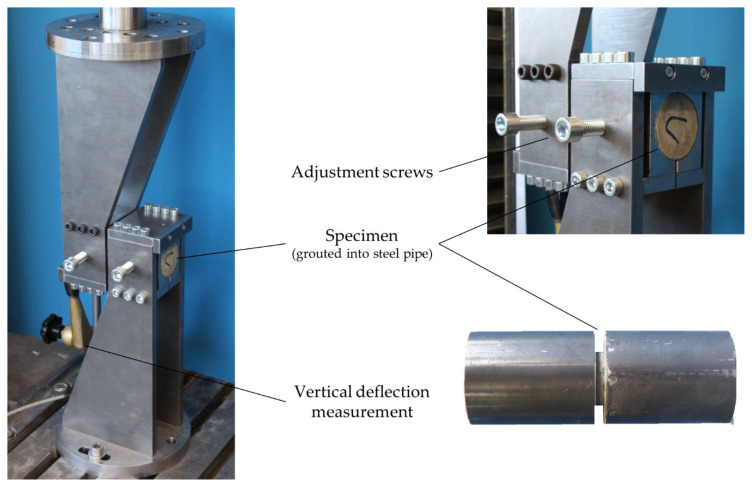
Proposed shear test setup for printed concrete specimen.

As described above additional shear tests on drill cores were conducted. Experimental characterization of shear fracture along sliding planes in casted concrete is a well-established area of research. In the last few decades, several different shear test setups have been proposed with the purpose of providing a pure state of shear stress along a ligament. Examples of existing testing methods are push-off tests by [38,39] as well as notched tensile tests by [40,41], a comprehensive overview is given in [42]. Even though proposed for mode II crack characterization, none of these setups produces a pure shear situation [43]. Either due to eccentric loading or because of the developing deformation during the test, the obtained shear fracture data is biased through an unintended contribution of a mode I tensile crack. Therefore, refined test setups suppressing the tensile stresses perpendicular to the tested material ligament have been proposed as exemplified by the modified Iosipescu test [44], which introduces compressive stresses perpendicular to the ligament at both notches and therefore suppresses tensile cracking. Another testing arrangement by [41] introduces a pure shear stress state in the ligament without significant normal stresses. Still, in both setups, a nonuniform shear stress distribution along the ligament length with high shear stress peaks at both ends of the ligament develops. As a consequence, specimens with shorter ligament length tend to deliver higher average shear fracture strength as reported by [43]. Obviously, the nonuniform shear stress distribution along the ligament length leads to a boundary effect that complicates an objective characterization of a representative material zone. Until now, there is no test setup at hand able to induce pure shear stresses with uniform distribution along a tested ligament. Currently available test setups suffer from the boundary effect, that falsifies the characterization of mechanical properties of a representative material zone. Based on this review of existing shear test methods, the authors developed a new setup to load drill cores, extracted from printed structures in shear, as shown in Figure 7.

The intention of this setup is, on the one hand, to provide approximately pure shear loading and, on the other hand, to reduce inadvertent size effects. To this end, the authors propose the use of specimens with small dimensions (29.5 mm diameter of drill core respectively interface area) leading to a rather uniform, constant shear stress distribution in the tested interface. With this test setup, we may reasonably assume to obtain average shear capacities that are close to the physical shear strength of the printed interface.

Before performing the tests, the drill cores are grouted into plane steel pipes with a gap of 4 mm. Inside the testing area the relevant interface is arranged with the respective interval times between 1 and 40 min as well as casted concrete. Through the plane steel pipes the specimens can be precisely mounted to the test rig. After inserting the specimen into the test rig, it is positioned by the semi-circular steel parts, which prevent a rotation (bending) of the specimens but allow free horizontal movement of the specimens (in longitudinal axis of drill core) and thus avoid restraining forces in the sheared-off interface.

An Instron 5582 universal tester was used as the testing device. The tests were driven displacement controlled with a rate of 0.2 µm/s. The test setup can be assumed stiff and the high size accuracy results in a normal force free support.

## 4. Experimental Results

### 4.1. Flexural Bending Tests (Unnotched)

**Figure 8:**
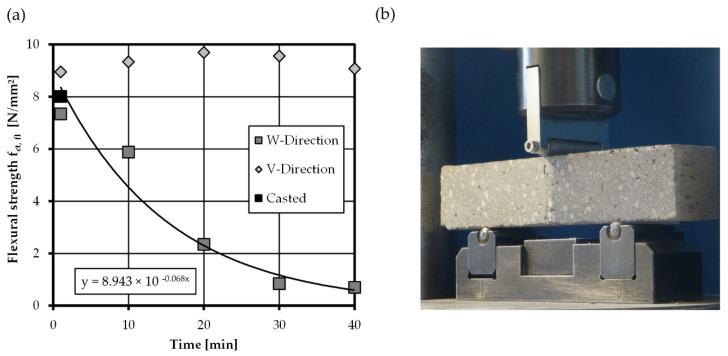
(**a**) Development of average flexural strength in W- and V-Direction with increasing interval time, note that the flexural strength of casted specimens is 8 N/mm^2^, (**b**) test setup flexural bending tests according to [37] (note the different concrete colors, marking the tested interface).

In the following, the results of flexural bending tests of printed interfaces are presented. Figure 8b shows the test setup to assess the anisotropic material behavior of printed concrete subjected to flexural loading. To investigate the adhesive properties, concrete with different colors was used in the layers adjacent to the tested interfaces. The flexural strength *f_ct,fl_* was determined from the measured bending moment. The results of the bending tests are shown in Figure 8a and illustrate the development of the average flexural strength of casted and printed specimens over time.

Perpendicular to the interface (W-orientation), only a slight decrease in flexural interface strength is found for 1 min interval time. After 10 min, a strength reduction of approx. 25% is observed and, from 20 to 40 min, the strength reduces severely to less than 25%, compared to casted concrete. The reduction curve can be described with a good fit by an exponential function, shown in Figure 8a. Results from Wolfs [24] gained within the same test setup showed a reduction in flexural strength of merely 20% within the first 4 h, but with the interfaces protected from drying during the interval times. Obviously, the own test results support the hypotheses, that dehydration of interfaces may be a major cause of strength reduction in printed structures.

**Figure 9:**
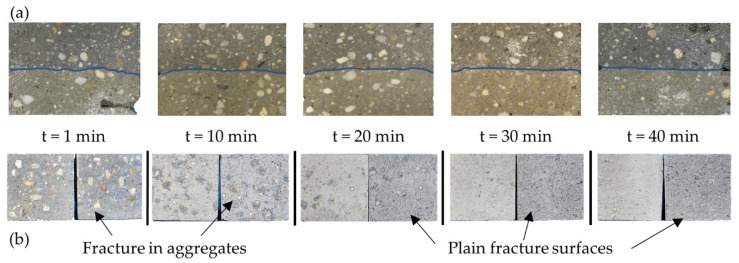
Quality of adhesion with increasing interval time—(**a**) undulation of interface region and (**b**) fracture surfaces after 3-point bending test.

This finding is also corroborated through visual analysis of fracture surfaces from 1 min to 40 min interval time in Figure 9. For an interval time of 1 min, the surface is rather rough with the fractures mainly running though the aggregates, see Figure 9b. In contrast, for an interval time of 10 min there are only few aggregate fractures, while some “dots” in the fractured surface indicate the adhesion of darker concrete. With increasing interval time, the occurrence of adhesive regions reduces rapidly, leading to rather plain surfaces at interval times of 30 min and beyond.

Through the different colors of the layers, it was also possible to analyze blending of concrete in subsequent layers as well as corrugation and undulation of the interfaces, Figure 9a. The visual evaluation reveals a severe reduction of material blending of subsequent filaments and decreased corrugation of interface for interval times larger than 10 min. Obviously, the dehydration process in combination with setting of concrete creates a skin effect that encounters blending and adhesion of adjacent layers, although the setting time is not reached by far.

The observations made in tests loaded in V-direction (stresses along interface) are in hard contrast to results in W-orientation. The development of flexural strength over time shown in Figure 8a indicates an increase in strength compared to casted concrete. No patterns can be observed in the progression of the values over the various time intervals. The calculation leads to an average flexural bending strength of 9.3 N/mm^2^ and an increase of around 16% compared to casted concrete. Results from Wolfs [24] authenticate the results and show that the V-direction does not react sensitive to dehydration or interval time. The strength increase can be explained through denser material packing inside the layers. Rahul et al. [27] found a decreasing porosity in the bulk material compared to casted concrete. This is due to the high pump pressure leading to a higher compaction of material. Since the stresses mainly act in the bulk material a correlation with increasing flexural strength or material properties is likely.

The characterization of the bending tensile strength with unnotched specimens is a frequently used standard test in several studies on digitally fabricated concrete. However, it is interesting to note that the flexural strength is not a material property in the strict sense, but describes a property dependent on both the mode I concrete behavior (strength and fracture energy) and the specimen geometry. This is why the flexural strength is only suitable for comparing results of specimens with identical geometry. A direct use of the flexural strength in the design of printed specimens is not useful, as this value is not objective but always influenced by the test specimen’s geometry.

### 4.2. Flexural Bending Test (Notched)

**Figure 10:**
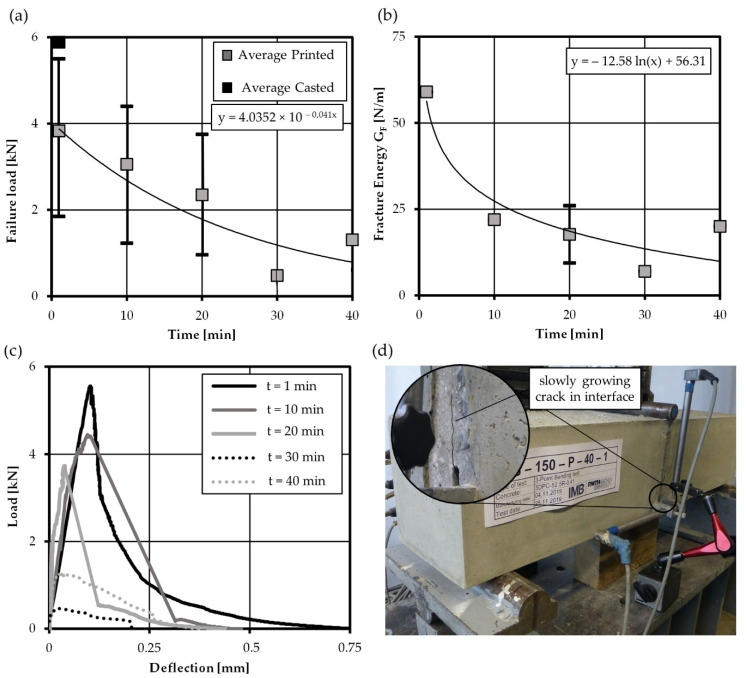
Development of (**a**) fracture load and (**b**) fracture energy over elapsed interval time, (**c**) exemplary load-deflection curves and (**d**) test setup with deflection measurement.

Further bending tests with hybrid specimen were conducted to characterize the mechanical properties of printed interfaces. Some tests were performed displacement controlled, which leads to slowly growing cracks in the interface region as illustrated in the test setup in in Figure 10d. Here, the fracture energy was determined by integrating the load-deflection curve and adding self-weight and weight of the test rig according to Hillerborg [30]. As shown in Figure 10a,b, a clear decrease in average fracture load and fracture energy was observed with increasing interval time. Compared to casted specimens, only a minor decrease for low time intervals was found, while it dropped significantly for interval times greater than 10 min. This matches investigations by Roussel [11] who also found a clear decrease in interface properties for specimens not protected from drying. As shown in the load-deflection curves, Figure 10c, the capacity of the interface to deform and transfer residual tensile stresses after the load-peak decreases. Accordingly, Figure 10b reveals a distinct decrease in fracture energy after 10 min interval time. In general, the presented notched bending test method is very easy to perform and gives reproducible results on fracture load and fracture energy of printed interfaces. However, the manufacturing effort is comparatively high, as different working steps (printing of interface region, casting of beam end zones) are necessary to produce the specimens.

### 4.3. Uniaxial Tensile Test

In addition, uniaxial tensile tests were conducted in a closed-loop deformation-controlled test setup to assess tensile strength and fracture energy of the printed interfaces.

**Figure 11:**
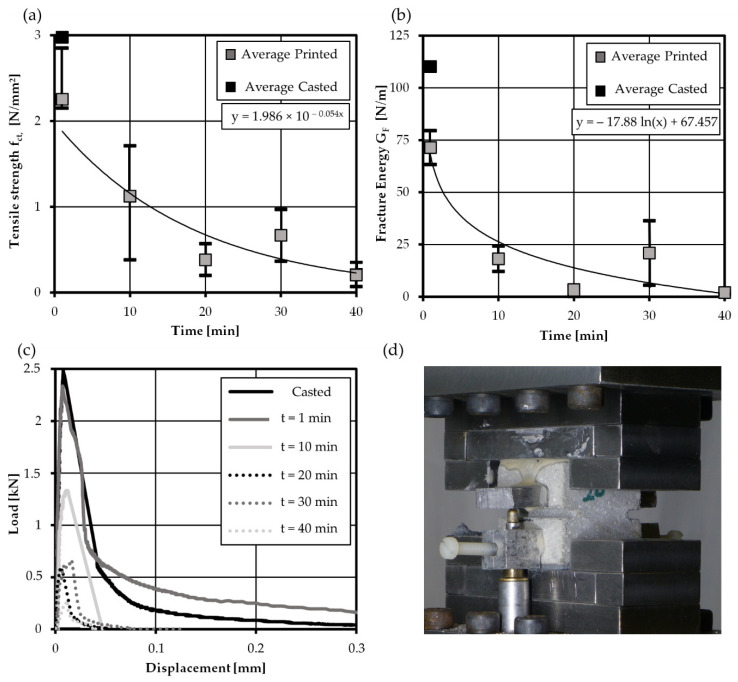
Results of uniaxial tensile tests—development of (**a**) average flexural strength and (**b**) average fracture energy with increasing interval time, (**c**) exemplary load deflection curves and (**d**) closeup-view on test setup.

Here, the fracture energy is determined through integration of post-peak load-displacement curve which corresponds to the load-crack opening response (without elastic elongation of concrete) [31,45]. Figure 11a reveals a decrease in tensile strength with increasing interval time. The influence in strength decrease between casted and printed specimens for an interval time t = 1 min is in a similar range as for bending beams, which verifies the consistency of both testing methods. For all time intervals larger than 10 min, the fracture energy drops significantly, as shown in Figure 11b, due to the decreasing tensile strength in combination with the more brittle post peak deformation behavior. So far, merely Wolf [24] investigated fracture energy of printed specimens by use of direct tensile tests with non-rotating plates, similar to the presented test setup. The average fracture energy of tests by [24] at an interval time t = 1 min was 28 N/m, which is rather low compared to own test results. This small fracture energy may be explained by the concrete mix design with small maximum grain size of 1 mm. For plain concrete, the maximum grain size has a decisive influence on fracture energy [29,31]. However, for 3D printed concrete, the influence of aggregate size on fracture energy has not yet been systematically investigated.

In order to assess the fracture energy for plain concrete a very stiff test setup is needed, as stated by Hillerborg [45]. Furthermore, a very precise installation of specimens without any eccentricity has to be guaranteed to avoid the introduction of inadvertent bending loads. Therefore, Hillerborg recommends the use of 3-point bending test with a reduced complicity to determine the fracture energy. However, for printed concrete a severe reduction of stiffness and fracture energy is observed. In consequence, also a less stiff setup may be sufficient to achieve a comparable stiffness ratio of specimen and test setup, compared to casted concrete. Therefore, the uniaxial tensile test seems a promising alternative to assess the fracture energy and tensile strength of printed interfaces.

### 4.4. Softening Hypothesis and Comparsion of Fracture Energy Results and Rest Setups

**Figure 12:**
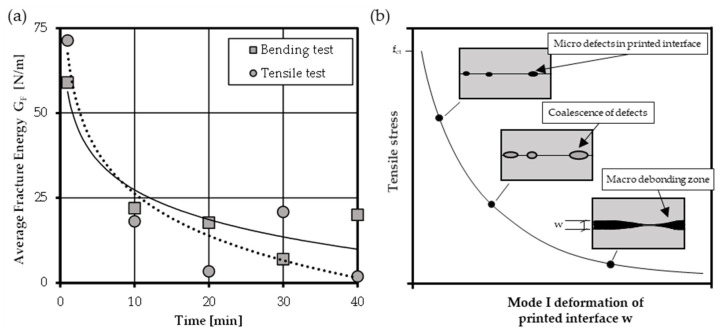
(**a**) Development of average values of fracture energy with increasing interval time—determined by uniaxial tensile tests and three-point bending tests and (**b**) softening hypotheses for printed cementitious materials.

In the following, a brief overview on the results and the strengths and weaknesses of the conducted test methods (direct tensile tests/notched bending test) regarding the determination of fracture energy mode I is given. In general, the average values of fracture energy for casted concrete reveal comparable results to similar concrete mixtures from literature. The notched flexural bending test and the uniaxial tensile test showed an overall good accordance and are both suitable for the determination of fracture energy as Figure 12a reveals. The results of both test setups point to a reduction in fracture energy due to the pure presence of a printed interface. In addition, both test series state a clear decrease in fracture energy for increasing interval times. The trend lines of both test setups show a good correlation and point to a very brittle failure behavior of printed interfaces for higher time intervals. Due to the longer interval times, drying of the surface is likely, which results in a decreased adhesion capacity. Through this, the crack path is predefined to evolve along the interface region which leads to a smooth and straight crack path.

In contrast to casted concrete, where a tortuous crack path through aggregates and matrix can be observed, microcracks develop along the interface with a given, predefined direction, as shown in Figure 12b. The authors believe that when exceeding the tensile strength, accumulations of micro defects occur in the interface zone. A quick coalescence of these defect results in debonding of the interface region, which is going along with an increasing brittleness in post-peak failure and reduced softening behavior compared to monolithic concrete.

As both test setups lead to comparable results, the decision to choose either the one or the other setup should be based on the test infrastructure at hand and on the effort of specimen production. Due to their hybrid nature, bending test specimens composed of casted and printed regions are rather difficult to produce. However, such tests are robust and less sensitive to inadvertent eccentricities. As the bending load induces a rather stable crack growth and propagation process in the notched specimens, these tests can be done in a setup with conventional deformation control. These tests do not necessarily have to be performed with a closed-loop testing procedure and there are no specific requirements regarding the stiffness of the test setup.

By contrast, the direct tensile tests have to be performed in a closed-loop testing procedure and in a very stiff testing frame to ensure a proper measurement in the post-peak branch. Proper installation without eccentricities is of crucial importance. However, it is an important advantage of these tests, that they can be performed either at easy-to-produce specimens (e.g., consisting of only two printed filaments) or at small samples extracted from larger 3D printed structures.

### 4.5. Shear Test

**Figure 13:**
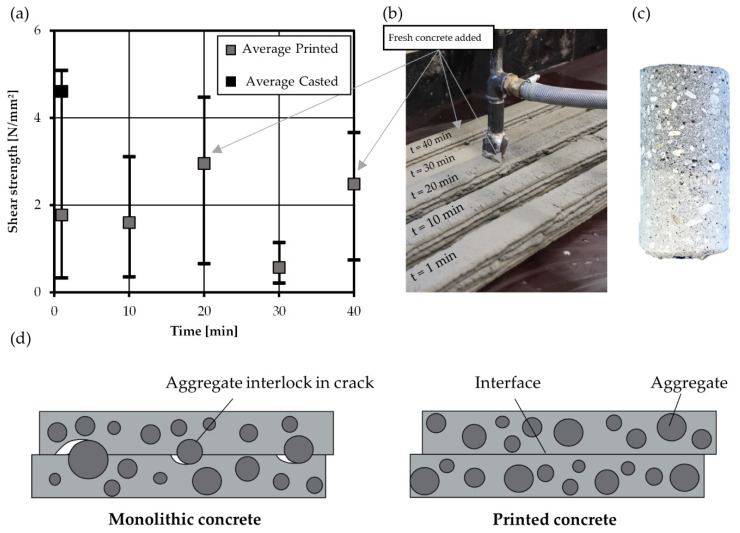
Results of shear test on printed specimen and values of (**a**) shear strength and (**b**) printing process and instants of adding fresh concrete (20 min, 40 min), (**c**) specimen and (**d**) aggregate distribution in monolithic and printed concrete.

Furthermore, shear tests were conducted using the test setup presented in Section 3.4.4. Generally, the shear test results had a comparatively high scatter, which may be due to the rather small dimensions of the tested ligament surface. The shear strength of casted reference tests was 4.6 N/mm², which is a in reasonable range compared to results of shear and push-tests from the literature. In contrast, there is a remarkable decrease in shear strength found for printed specimens in the interface region (approx. 1.8 N/mm² for 1 min interval time). In contrast to monolithic concrete where shear is transferred by a combination of intergranular compression and perpendicular tensile stresses, the printed interface represents a discrete perturbation. Here, shear is solely transferred via adhesive properties of concrete. Due to the layering of material, the interface region contains only cement matrix which excludes the existence of any beneficial structural effects like intergranular compression or aggregate interlock (after the formation of a discrete crack). After reaching the adhesive shear strength of the cement matrix, a crack emerges along the printed interface leading to sudden fracture of the specimens, as shown in Figure 13d. Thus, shear failure of printed interfaces is a very brittle process. Though testing with the deformation controlled and using an almost rigid test setup, it was not possible to record any post-peak residual shear stress transfer. Thus, mode II fracture energy of printed specimen seems to be negligibly small.

In addition to the aforementioned tests, also the effect of interval time (1 min to 40 min) has been investigated. However, these tests did not deliver reliable results. Due to problems in the concrete mixing procedure, fresh concrete was added during the printing event (approx. at interval times of 20 and 40 min, see Figure 13b). This led to an increased moisture content in the interface and enhanced shear strength at these interval times. Thus, the results of the shear test are not consistent with the rest of the experimental campaign, where the concrete age was chosen equal to the interval time. In addition, it cannot be excluded that drilling of the small sized specimen—though performed with great care and caution—may have caused some mechanical damage to the specimens prior to testing. Especially, a test setup without drilled specimen, like the TORAX setup (chapter 5.) could help to eliminate such inadvertent effects. Based on the measured results, it is not possible to judge the effect of interval time on shear strength. Further investigations are required to assess the influence of fresh concrete and interval time on fracture process under shear and mixed mode actions. However, the authors expect a decreasing shear strength with increased interval time, similarly to what has been observed for tensile and bending behavior.

### 4.6. Compressive Tests

**Figure 14:**
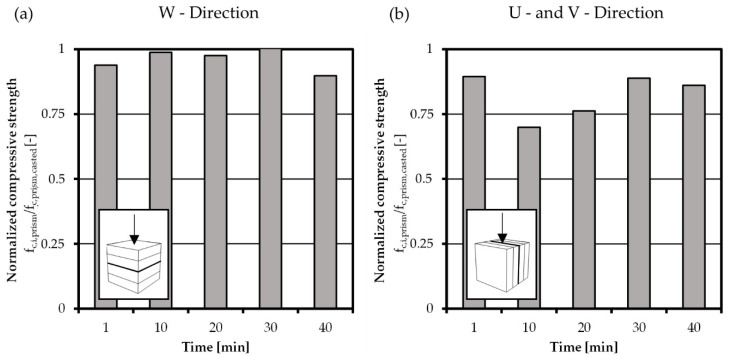
Development of compressive strength of printed specimens in (**a**) W-direction and (**b**) V-direction, with the respective arrangement of interfaces regarding the load direction.

To investigate the anisotropic behavior of printed concrete subjected to compressive stress, compressive tests in V- and W-direction were conducted. The behavior in U-direction is represented by the V-direction as shown in Figure 14b. As a testing device a Piston Stroke TT1184 with a 300 kN load cell was used to test halves of the previous tested flexural bending prisms with a 40 × 40 mm^2^ load application area. For the load application rigid plates without transversal freedom were used. Per interval time and direction four values were measured.

The average compressive strength of the used casted concrete after 22 days was determined to 65.8 N/mm². In order to show the anisotropic behavior of the printed concrete the results with increasing interval time in the different directions are normalized to the average compressive strength of casted concrete and shown in Figure 14a,b. In general, no time dependent behavior for the different directions is visible, but an anisotropic behavior is found. This is expressed in the comparison of the average compressive strength of all time intervals. While the compressive strength perpendicular to the interface (W-direction) is in average 96% of casted concrete, the strength in V- and U-direction is only 82% in average.

## 5. Torsion Test Setup for Mixed Mode Testing

Generally, the results gained from the aforementioned test setups showed good accordance and provide a satisfactory overview of mechanical performance of 3D printed concretes and mortars. However, obviously, many different specimen formats, manufacturing methods (fully printed/ hybrid) as well as different test setups fulfilling specific requirements each (e.g., with regard to stiffness, force- or displacement loading, open or closed-loop control) need to be consulted. This is time- and cost-intensive and bears the potential for errors. In addition, the coherence and comparability of the results obtained in different test rigs is not easy to prove. A uniform testing strategy based on a universal specimen format and test setup, eligible to characterize all relevant mechanical properties of hardened 3D printed concrete, is lacking, so far.

**Figure 15:**
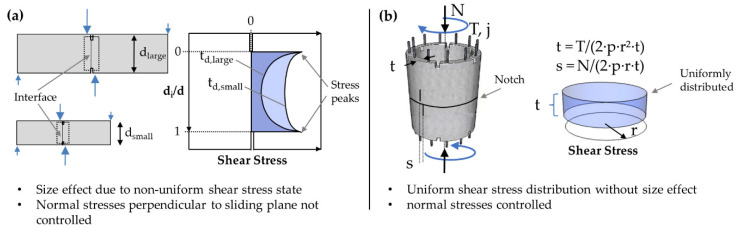
Comparison of shear profiles in the test zones of the (**a**) conventional notched rectangular shear test specimen and (**b**) tubular twisted specimen with uniform shear profile in the circumferential direction.

In addition to coherence and reproducibility, an important requirement that has received little attention in the past is the scalability of the results. In conventional shear tests with finite shear face length non-uniform shear stress distributions with stress peaks at both edges of the ligament occur leading to size effects (Figure 15a). This is why, these setups are debatable. In contrast, the previously proposed shear test setup in this article (Figure 7) is capable of generating almost constant shear stresses in the sheared-off interface and thus to reduce size effects. However, due to the use of a very short filament length, it also tends to produce a high scatter of test results when applied to printed concrete, because either the presence or absence of defects in the small sheared-off interface has significant influence.

Obviously, there is a strong need for a universal test setup, that allows:

- coherent characterization of all relevant mechanical properties of 3D printed concrete and mortar (compressive, tensile, shear strength as well as fracture energy);
- use of a uniform test specimen with standardized geometry and production process/ printing procedure;
- testing with a high degree of reproducibility and minimized scatter;
- as well as avoidance of inadvertent size effects to gain objective test results.

To overcome the shortcomings of current test setups and fulfill these requirements, the authors propose a new testing procedure that can simultaneously load a specimen in torsional and axial directions (Figure 15b). The general idea of torsion testing to characterize shear behavior of materials is well-known, e.g., from tests on adhesive joints [46] and from own test campaigns performed on small-scale shear connectors between steel and concrete [47]. To prove the technical feasibility of the proposed method for testing of concrete the applicants have conducted several tests on small casted concrete tubes under monotonic and cyclic loading [48]. During the tests, notched thin-walled concrete tubes were twisted while simultaneously controlling the normal stresses in the ligament. The obtained results proved the uniformity of strain distribution along the ligament. As the axial load and torsional moment can be controlled independently from each other, the test setup allows for a systematic variation of deviatoric and volumetric strains within the ligament and thus mode I, mode II as well as mixed mode loading. Currently, a new combined torsion-axial force testing machine (TORAX) with 2 MN axial force and 1 MNm torsional moment is being built up at the institute’s lab which will allow us to upscale the testing method to 3D printed specimens even with large dimensions.

The developed experimental method exploiting the macroscopically continuous strain and stress profiles in the circumferential direction of a tubular specimen is ideally suited for the characterization of 3D printed concrete specimen. It provides a fine resolution control over the dosage of either shear, tension or compression within a tested zone and can thus be used to closely monitor the local strain and stress redistribution within the material structure during the loading. This unique possibility shall be systematically exploited in the future to obtain a deep and general insight into the phenomenology of damage and failure within the material structure of 3D printed concrete.

## 6. Conclusions

Within the scope of this paper, special testing methods designed towards the requirements of 3D printed concrete have been developed and evaluated. By initially implementing a printing system for additive manufacturing of concrete, different specimen types were produced. By the help of different testing setups, the influence of concrete age and interval time on the mechanical properties were investigated. Therefore, different testing series with interval times from 1 to 40 min were conducted. It should be noted that the investigated interval times were set equal to the concrete age after mixing of concrete at the beginning of a printing event. It is obvious that continuous mixing improves the material properties and ensures consistent fresh concrete properties, but also requires enhanced machinery. Therefore, the findings made in this paper are a conservative (lower bound) approximation of mechanical properties of hardened 3D printed concrete and mortars.

Based on the gained results, following conclusions on testing scheme and mechanical properties of printed concrete are made:

- Despite using different specimen dimensions and testing methods, the performance of interfaces with regard to strength and fracture energy showed a uniform phenomenological behavior.
- Strong anisotropy of printed concrete has been observed for all different loading cases (tension, bending, shear, compression). This anisotropic behavior has to be taken into account in the development of design and engineering models.
- The interface properties under mode I (tensile and bending) and mode II (shear) action decrease significantly with increasing interval times as dehydration of interface area impedes adhesion of subsequent filaments. The reduction in strength can be well approximated by an exponential curve.
- The fracture energy reacts sensitive to the presence of interfaces, too. Reduced aggregate content in interface area and dehydration due to increasing interval times heavily reduce fracture energy and consequently the ductility and toughness of printed concrete. The decrease in fracture energy can be approximated by a log-function.
- By contrast, the interval time did not significantly affect the performance of 3D printed concrete under compressive loading. Here, still the anisotropic behavior is of crucial importance (3D printed concrete performs well under compressive stresses vertical to printing path and shows a splitting failure with decreased strength when loaded in the direction of the printed interfaces).
- Especially determination of fracture energy is a tricky task. It can be determined either by uniaxial tensile tests or bending tests. Experimental procedures to be applied should be selected based on the testing infrastructure at hand. If only displacement-controlled testing setups (without closed-loop control) are available, a 3-point bending test provides stable results. Such tests are not very sensitive to eccentricities but require rather large test specimen. In contrast, uniaxial tensile tests need a very stiff test setup with closed-loop control and accurate installation without eccentricities. These tests allow determination of fracture energy and tensile strength at small (and easy-to-produce) specimen.
- For printed concrete shear stress is solely transferred via adhesive properties of cement matrix which results in reduced properties compared to casted concrete. Further investigations on the influence of interval time on shear strength are required.
- A new uniform testing procedure based on combined torsion-axial force (TORAX) test setups have been proposed for coherent characterization of all relevant mechanical properties of 3D printed concrete and mortar (compressive, tensile, shear strength as well as fracture energy).

Although current concrete design models do not explicitly use tensile strength and fracture energy as input values, this should not obscure the fact that these models implicitly rely on the existence and presence of a specific amount of both mechanical properties. Therefore, the results of the presented test series, showing a severe reduction of this mechanical properties, should be taken into account, when adapting current or developing new design tools for 3D printed concrete structures.

## Figures and Tables

**Table 1:** Composition of concrete and sieve fraction.

Component	“Heidelberg” CEM I 52,5 R ^1^	“Powerment” Fly Ash	Water	Aggregate
Amount (kg/m³)	550	250	280	1172
**Sieve Fraction (mm)**		**Ratio Aggregate (%)**			
0.0–0.2		25			
0.2–1.0		30			
1.0–2.0		25			
2.0–4.0		20			

^1^ Production plant Enningerloh Süd, Germany.

**Table 2:** Specimen dimensions regarding testing method and target value.

Testing Method	Investigated Parameters	Specimen Dimensions	Notch
Three-point bending (Unnotched)	Flexural strength/ Anisotropy	40 × 40 × 140 mm³	-
Three-point bending (Notched)	Fracture Energy mode I	150 × 150 × 700 mm³	30 mm
Uniaxial tensile test	Tensile strength / Fracture energy mode I	40 × 40 × 40 mm³	7 mm
Compressive test	Compressive strength/ Anisotropy	40 × 40 × 40 mm³	-
Shear test	Shear strength	Drill core Ø 29.5 mm	-

## Data Availability

Data contained within the article. Further data available on request, due to ongoing research
